# Tasco^®^: A Product of *Ascophyllum nodosum* Enhances Immune Response of *Caenorhabditis elegans* Against *Pseudomonas aeruginosa* Infection

**DOI:** 10.3390/md10010084

**Published:** 2012-01-11

**Authors:** Saveetha Kandasamy, Wajahatullah Khan, Franklin Evans, Alan T. Critchley, Balakrishnan Prithiviraj

**Affiliations:** 1 Department of Environmental Sciences, Nova Scotia Agricultural College, P.O. Box 550, Truro, NS, B2B 5E3, Canada; Email: skandasamy@nsac.ca; 2 Genome Research Chair Unit, Department of Biochemistry, College of Science, King Saud University, PO Box 2455, Riyadh 11451, Saudi Arabia; Email: wkhan@ksu.edu.sa; 3 Acadian Seaplants Limited, 30 Brown Avenue, Dartmouth, NS, B3B 1X8, Canada; Email: fevans@acadian.ca (F.E.); alan.critchley@acadian.ca (A.T.C.)

**Keywords:** Tasco^®^, *Ascophyllum nodosum*, *Caenorhabiditis elegans*, *Pseudomonas aeruginosa*, virulence factors, innate immunity, gene expression, quorum sensing

## Abstract

The effects of Tasco^®^, a product made from the brown seaweed (*Ascophyllum nodosum*) were tested for the ability to protect *Caenorhabditis elegans* against *Pseudomonas aeruginosa* infection. A water extract of Tasco^®^ (TWE) reduced *P. aeruginosa* inflicted mortality in the nematode. The TWE, at a concentration of 300 µg/mL, offered the maximum protection and induced the expression of innate immune response genes *viz.*; *zk6.7* (*Lypases*), *lys-1* (*Lysozyme*), *spp-1* (*Saponin like protein*), *f28d1.3* (*Thaumatin like protein*), *t20g5.7* (*Matridin SK domain protein*), *abf-1* (*Antibacterial protein*)* and f38a1.5* (*Lectin family protein*). Further, TWE treatment also affected a number of virulence components of the *P. aeuroginosa* and reduced its secreted virulence factors such as lipase, proteases and toxic metabolites; hydrogen cyanide and pyocyanin. Decreased virulence factors were associated with a significant reduction in expression of regulatory genes involved in quorum sensing, *lasI*, *lasR*, *rhlI and rhlR*. In conclusion, the TWE-treatment protected the *C. elegans* against *P. aeruginosa* infection by a combination of effects on the innate immunity of the worms and direct effects on the bacterial quorum sensing and virulence factors.

## 1. Introduction

*Ascophyllum nodosum*, a large brown alga commonly known as “Rockweed”, is found in the cold waters of the North Atlantic Ocean [1]. *A. nodosum* is widely used in the agricultural and horticultural industries [2,3,4]. It has also been used as an animal feed supplement due to antioxidant properties and improvement of stress tolerance and immune function in animals [5]. A commercial *A. nodosum* animal feed supplement known as Tasco^®^ is reported to improve the health of animals by modulating gastro-intestinal (GI) tract microflora, thereby increasing stress resistance through activation of the immune system [6]. Recently, we have observed that the water extract of Tasco^®^ (TWE) imparts thermal stress tolerance in invertebrate animal model *Caenorhabiditis elegans*. Our results showed that TWE at a concentration of 300 µg/mL significantly enhanced thermal stress tolerance as well as extended the life span of *C. elegans* [7]. 

*A. nodosum* contains several bioactive compounds such as polysaccharides (e.g., alginic acid, fucans, laminarin), polyunsaturated fatty acids (PUFA), vitamins, antioxidants, peptides, secondary metabolites (e.g., phlorotannins) *etc.*, [1,8,9]. These compounds are known to possess beneficial properties such as antioxidant, anti-bacterial, cell-mediated immunomodulation [10,11] and anti-inflammatory activities [12]. The antibacterial activities of *A. nodosum* extracts against several pathogenic bacteria, e.g., *Escherichia coli*, *Pseudomonas*, *Micrococcus*, *Aerobacter*, *Brucella*, *Salmonella*, *Klebsiella and Streptococcus* were demonstrated by Vacca and Walsh [13]. The sulphated polysaccharides are an important group of sugars that occur in marine macroalgae, the quantity vary amongst red, brown and greens. Fucose containing polymers (FCPs) and laminarins are the major sulphated polysaccharides (SPs) of brown algae, while carrageenans and ulvan are found in red and green algae, respectively [14]. The dry matter of *A. nodosum* contains 1–7% laminarin, 4–10% fucans and 25–30% alginates. The FCPs in *A. nodosum* include considerable amounts of L-fucose and sulphate ester groups [2,15].

The ubiquitous bacterium, *Pseudomonas aeruginosa*, is an opportunistic human pathogen causing widespread lung infection in cystic ﬁbrosis (CF) patients [16]. *P. aeruginosa* may also cause serious infection in immune compromised, HIV and cancer patients [17]. Pathogenesis of *P. aeruginosa* is largely mediated by secretory proteins (*i.e.*, elastase, alkaline protease and lipases) and secondary metabolites (e.g., pyocyanin, pyoverdine and hydrogen cyanide). *P. aeruginosa* colonies are known to form tenacious biofilms which are thought to enhance successful infection in humans and in animals [18,19]. This bacterium produces a redox-active phenazine compound called pyocyanin (1-hydroxy-5-methyl-phenazine). It is a characteristic chloroform-soluble, blue pigment that kills higher animal cells by generating reactive oxygen species (ROS) and arresting their cellular respiration [20]. 

Hassan and Fridovich [21] and Hassett and Cohen [22] described a mechanism for the toxicity of pyocyanin whereby electron flow from biological pathways was diverted to increase the production of intracellular oxygen reduction products, ultimately leading to cell death. Pyocyanin shows strong antibiotic activity against a wide variety of microorganisms, thereby suppressing competing microorganisms [21,23,24]. Biosynthesis of cyanide has been demonstrated as one of the primary components in microbial secondary metabolism and it is well characterized in *P. aeruginosa* pathogenesis. The production of hydrogen cyanide (HCN) occurs during late log and early stationary phases of' bacterial growth [25]. 

Bacteria communicate through an extensive array of extracellular signal molecules. Production and secretion of these mediate cell-to-cell communication which coordinates the expression of various genes within the bacterial population and aids in the formation of biofilms in response to specific environmental or physiological conditions [26,27]. The process of sensing individual cells by the accumulation of diffusible, low-molecular-weight signal molecules is known as “quorum sensing” (QS) [28]. There are two well-deﬁned QS systems identified in *P. aeruginosa* namely *las* (mediated by transcriptional activators *lasR* and *lasI*) and *rhl* (mediated by the transcriptional activators, *i.e.*, *rhlR* with *rhlI*). An understanding of QS has opened a potential new avenue for treating bacterial infections.

*P. aeruginosa* stain PA14 is a clinical isolate that infects the model nematode *C. elegans*. This pathosystem was extensively studied by Tan *et al.* in [29]. An advantage of using this model is that it is genetically tractable because of the availability of a genome map for both the organisms and can be readily used as reference model for mammalian bacterial pathogenesis [29,30,31]. This pathosystem has been used to screen for anti-infective and antimicrobial agents, which have potential use in animal and human infections [32].

In this paper, we used the *C. elegans*-PA14 pathosystem to study the protective effects of TWE against *P. aeruginosa* infection. Furthermore, we studied the direct effect of TWE on the secreted virulence factors, biofilm formation and quorum sensing of *P. aeruginosa*.

## 2. Results

### 2.1. The *C. elegans* Killing Assay

**Figure 1:**
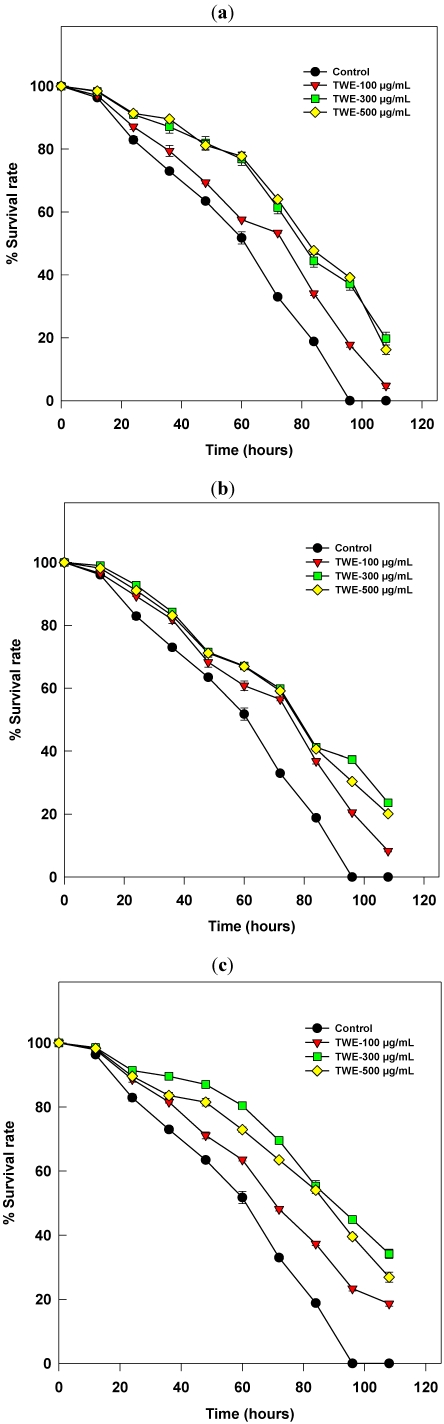
Effect of concentration of water extract of Tasco^®^ (TWE) on the death of *C. elegans* by the bacterial pathogen *P. aeruginosa*-strain PA14. Three different concentrations of TWE (100, 300, 500 mg/mL) were used in all the three experiments. (**a**) The dietary supplemented worms are exposed to bacterial lawn cultured without TWE-treatment; (**b**) The worms cultured on plain Nematode Growth Medium (NGM) without dietary supplementation were exposed to a bacterial lawn cultured with different concentrations of TWE; (**c**) The worms with a TWE-supplemented diet were exposed to a bacterial lawn cultured with different concentration of TWE.

**Figure 2:**
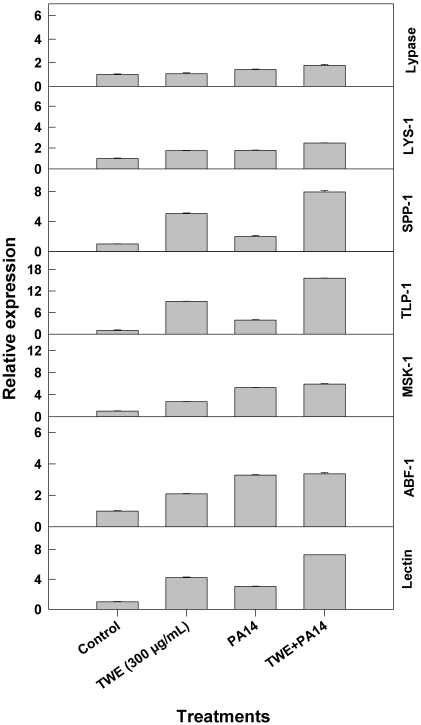
Relative expression of immune response genes in TWE-treated *P. aeruginosa*, strain PA14 as compared to the control.

**Figure 3:**
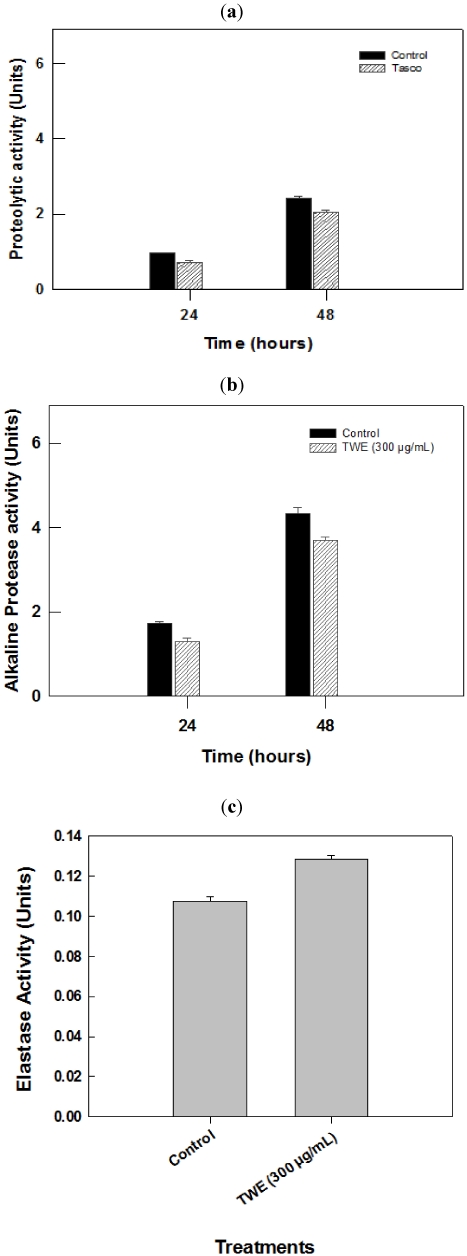
Effect of TWE-treatment on *Pseudomonas aeruginosa* secreted virulence factors. (**a**) Effect of TWE on the proteolytic activity of *P. aeruginosa*, strain PA14; (**b**) Influence of TWE on alkaline protease activity of *P. aeruginosa*, strain PA14; (**c**) Effect of TWE on the elastase enzyme activity of *P. aeruginosa*, strain PA14.

In general, the killing of *C. elegans* was much reduced in the TWE treatments in all three experiments (See Figure 1, Figure 2, Figure 3). There were no statistically significant differences observed in reducing the killing activity between 300 and 500 µg/mL in all the three experiments. However, activity was reduced at higher concentration (*i.e.*, 500 µg/mL), when compared to the optimum concentration (*i.e.*, 300 µg/mL). The effect was significantly less at the minimum concentration (100 µg/mL), when compared with the optimum. All worms were killed in the control treatment by the 96th hour of their exposure to the pathogen infection. However, a significant rate of survival (*p* < 0.05) was recorded with the TWE-treatments, even after 96 hrs. The reduced rate of killing was observed in the experiment conducted with TWE-supplementation in the culture plates of both *C. elegans* and PA14 instead either of previous treatments (Figure 3). Since 300 µg/mL showed a significant (*p* < 0.05) effect on reducing killing of the worms, we used the same concentration in all our further experiments. We did not observe any developmental abnormalities or death of *C. elegans* when the worms were fed with *E. coli* OP50, the usual laboratory worm diet along with the optimum concentration of TWE (300 µg/mL) (data not shown) [7].

### 2.2. Effect of TWE-Treatment on Immune Responsive Gene Expression of *C. elegans*

Dietary supplementation of TWE showed noticeable alterations in the expression of the immune response genes in *C. elegans*, when the worms were infected with the bacterial pathogen *P. aeruginosa*, strain PA14. We tested seven genes from the immune response pathway and observed that the levels of expression of all of the genes were significantly higher in the TWE-treatments as compared to the control. We observed that the *tlp* gene expression was about 18 fold more, following TWE-treatment than the control. Similarly, the *lectin* gene expression was increased 8 fold, *abf*-1 gene expression was increased 3 fold, *msk-1*gene expression was up-regulated 5 fold, *ssp*-1 gene expression was increased by the factor of 7, *lys*-1 gene expression was increased 3 fold, and the gene expression of *lypases* gene were increased by 2, following TWE-treatment, as compared to the respective controls (Figure 2). 

### 2.3. Effect of TWE-Treatment on *P. aeruginosa* Secreted Virulence Factors

Secretory virulence factors such as protease, alkaline protease and elastase were measured in the culture filtrate of *P. aeruginosa* cultures grown in nutrient broth supplemented with TWE. The results showed that the protease activity increased exponentially until bacterial growth reached the stationary phase. In general, there was an increase in enzyme activity in all treatments, including the control, with respect to time. It was observed that proteolytic enzyme activity was reduced (*p* < 0.05) from 0.75 to 0.5 units (after 24 hours), and 2.2 to 1.8 units (after 48 hours) in the TWE-treated cultures, as compared to the respective controls (see Figure 3a). A similar trend in reduction (*p* < 0.05) was also noticed with alkaline protease activity due to TWE-treatment (Figure 3b). However, the elastase enzyme activity in TWE-treated *P. aeruginosa* culture filtrates showed increased enzyme activity (*p* < 0.05) when compared to the control (Figure 3c). 

### 2.4. Effect of TWE on *P. aeruginosa* Toxic Metabolites

**Figure 4:**
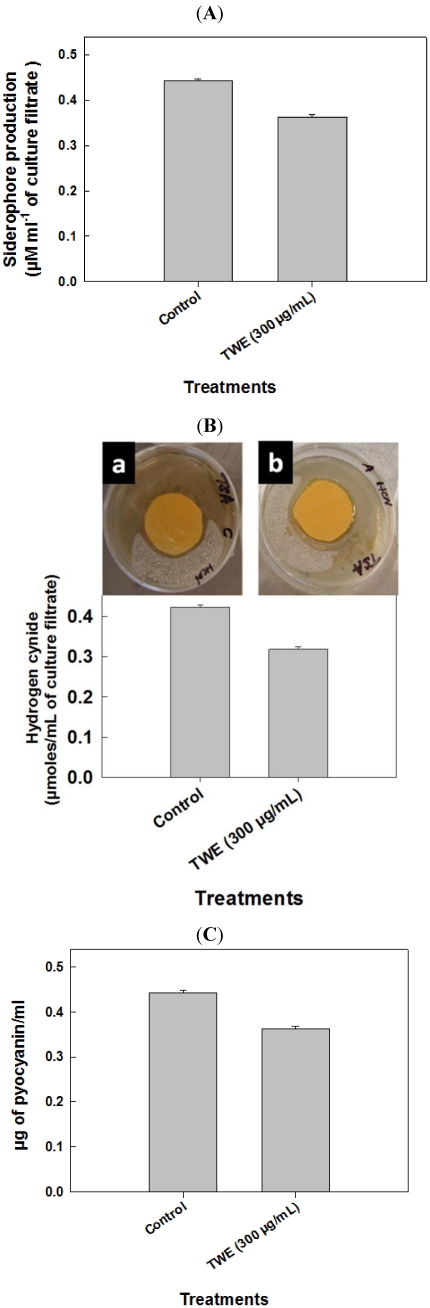
Effect of TWE on *P. aeruginosa* toxic metabolites. (**A**) Influence of TWE on the production of pyocyanin by the human pathogen, *P. aeruginosa*-strain PA14 (**B**) Effect of TWE on *P. aeruginosa*, strain PA14 hydrogen cyanide production: (**a**) Control; (**b**) TWE 300 µg/mL treated; (**C**) Effect of TWE on the production of the important secondary metabolite, siderophore by *P. aeruginosa*, strain PA14.

The pathogenicity of *P. aeruginosa* has been associated with the production of numerous virulence factors [18,19]. To determine the effect of TWE on the production of prime virulence factor, pyocyaninin the *P. aeruginosa*, strain PA14, after 48 hour culture, the organic fraction of pyocyanin was extracted in acidified water and quantified spectrophotometrically. The OD (520 nm) reading obtained was converted in to µg units, based on standard curve values. The results showed that the amount of pyocyanin production was 0.37 µg/mL in the TWE-treated culture, as compared to 0.45 µg/mL in the control (see Figure 4a). Production of HCN was quantified using a quantitative picrate assay. The HCN production was reduced (*p* < 0.05) in the TWE-treated cultures (0.32 µmoles/mL), as compared to control (0.43 µmoles/mL) (Figure 4b). Siderophore production was also much reduced (*p* < 0.05) due to TWE-treatment (Figure 4c). 

### 2.5. Effect of TWE on *P. aeruginosa* Bioﬁlm Formation

**Figure 5:**
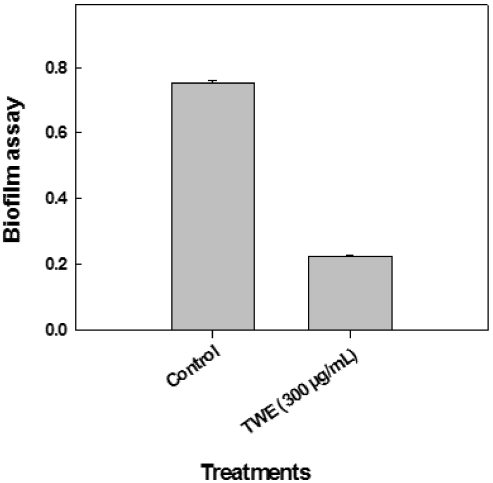
Influence of TWE-treatment on *P. aeruginosa* strain PA14 biofilm formation.

The TWE-treatment demonstrated that it significantly reduced (*p* < 0.01) biofilm formation by a factor of 4 when compared to the control (Figure 5).

### 2.6. Effect of TWE on Expression of *P. aeruginosa* Quorum Sensing Genes

The *las* and *rhl* quorum-sensing systems were studied since both play critical roles in *P. aeruginosa* pathogenicity, including synthesis and regulation of other important virulence genes of the QS biosynthetic pathways, as well as production of secondary metabolites believed to be toxic. It was observed that the growth and cell density of *P. aeruginosa* were reduced considerably following TWE-treatments (data not shown). 

**Figure 6:**
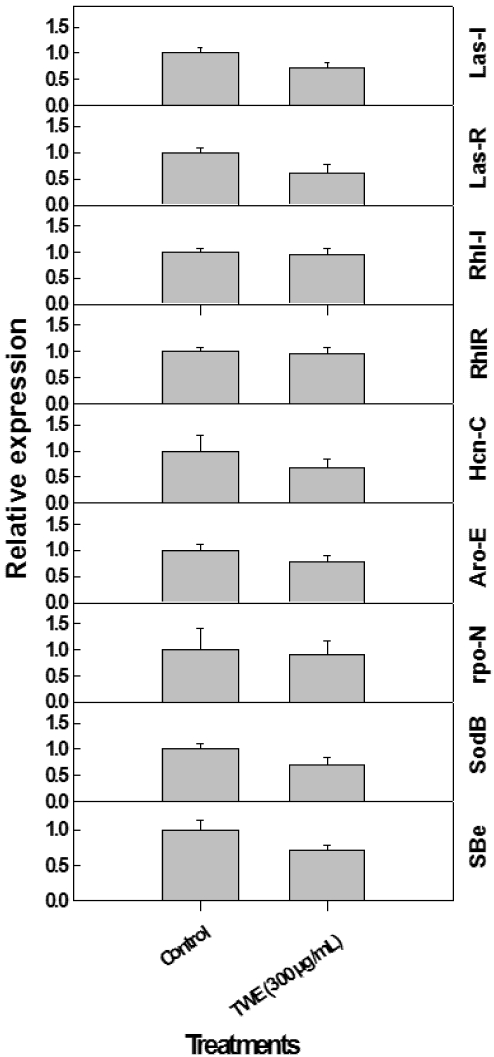
Relative expression of quorum sensing and virulence factor related genes in *P. aeruginosa* treated TWE.

To validate the results from the biochemical analysis of secondary metabolite production following TWE-treatment, gene expression studies were conducted using Real Time- PCR (RT-PCR). The results showed that the relative expression of major QS (*i.e.*, *lasI*, *lasR*, *rhlI and rhlR*) and virulence factors (*i.e.*, *hcnC*, *aroE*, *rpoN*, *sbe*, *sodB*, *phz*, *pyoS3a*) were considerably reduced due TWE-treatment over the control. The observed reduction in the gene expression was about two fold in TWE-treated cultures (Figure 6).

### 2.7. Antimicrobial Susceptibility Testing

**Figure 7:**
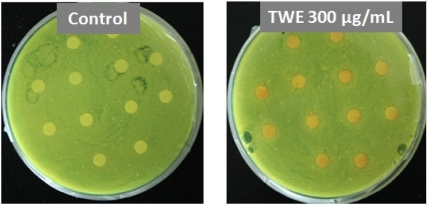
Effect of TWE on direct antimicrobial activity against *P. aeruginosa*.

Direct antimicrobial activity was observed by placing paper discs pre-treated with different concentrations of TWE on petri plates cultured with *P. aeruginosa*. The plates were examined at regular intervals for 3 days. It was established that TWE did not exhibit an antimicrobial effect on *P. aeruginosa* directly (Figure 7).

## 3. Discussion

*C. elegans* is a well-defined laboratory model to study the origin, function and evolution of innate immunity in higher animals. Modulations in gene expression are an important part of host-innate immunity, when pathogens are encountered. The innate defense system includes cellular immune responses (e.g., apoptosis, encapsulation, phagocytosis and nodule formation), up regulation of the prophenoloxidase system and synthesis of a cascade of both antimicrobial and antiviral peptides. A well-established *C. elegans*-*P. aeruginosa* infection model is available to demonstrate both fast and slow killing [33,34]. Using the established *C. elegans*/*P. aeruginosa* slow killing infection model, the present study tested the ability of TWE to induce immune responses in *C. elegans* and also any direct antibacterial potential against the pathogenic *P. aeruginosa* strain PA14. We observed a considerable reduction in the killing rate of *C. elegans* following TWE-treatment as compared to all controls used. These observations are in agreement with previous reports, where *A. nodosum* extracts have been demonstrated to significantly reduce coliform bacteria in the ileum and caecum [13], which in turn improved the immune system and boosted the immunity and antibacterial potential of the animal [35]. It was also reported that *A. nodosum* reduced the toxicity and enhanced the immunity of endophyte-infected Tall Fescue and has been found to improve the shelf life of processed beef significantly [36].

Tasco^®^ has also been shown to act as a biostimulant in the stress-induced livestock and involve in improving nutrient uptake, immune function and antioxidant activity [37]. Supplementation of cattle diet with *A. nodosum* has significantly reduced the fecal shedding of *E. coli* [38]. In agreement with this study, *A. nodosum* commercial extract, has been shown to reduce the number of colony forming units of *P. aeruginosa* [13].

Conserved signal transduction in *C. elegans*, in response to pathogen resistance, depends mainly on four important regulatory pathways; *i.e.*, (i) the p38 MAP kinase pathway; (ii) programmed cell death pathway; (iii) TGF-β pathway; and (iv) DAF-2 insulin/IGF-I like signaling pathway. The current study was aimed to analyze the effect of TWE on the immunity of *C. elegans* against the pathogen-*P. aeruginosa*, by analyzing the expression of selected innate immune response genes involved in various signaling pathways. *P. aeruginosa* infection uses host effector molecules along with pathogen virulence factor to suppress the immune function in *C. elegans*. It activates DAF-2 insulin signaling pathway, which translocate the DAF-16 proteins from nuclei leads down regulation of immune responsive transcriptional targets (*lys-1*, *spp-1*, *tlp-1*, *abf-1* *etc*.) [39]. The results from the present study showed that TWE had a greater influence on the increased levels of expression of the immune response genes (*lectin*, *abf-1*, *msk-1*, *ssp-1*, *lys-1* and *lypases*). A significant difference was the level of expression of *tlp* gene as compared to the control and all other genes examined in this study. These results clearly indicate that TWE is involved in the suppression of pathogen mediated activation of DAF-2 signaling pathway.

Brown algae contain a considerable number of potentially useful bioactive compounds [40]. Some of these bioactive compounds such as, sulphated polysaccharides (SPs) (e.g., fucose containing polymers), laminarin, phlorotannins and diterpenes present in the seaweeds have potential applications for their antimicrobial, immune-modulating and antioxidant properties. Reilly *et al.*, [6,41] reported that SPs significantly reduced problems in piglet weaning due to interactions with the microfloral populations of their guts. SPs are also known as free-radical scavengers and antioxidants which can be involved in prevention of oxidative damage and also a wide range of common, age-related diseases and degenerative conditions in living organisms [42]. An extract from the red seaweed, *Gracilaria tenuistipitata* showed a slight decrease in peroxidase activity, but an increase in lysozyme and antioxidant activities in the white shrimp (*Litopenaeus vannamei*), against the *white spot syndrome virus* (WSSV) infection [43].

Treatment with hot-water extract of the brown alga, *Sargassum duplicatum* also increased immunity and resistance in cultured shrimps (*L. vannamei*) [6]. Similar observations have been reported by other researchers, in various systems (see [44,45,46,47]). In addition, the oral introduction of a fucoidan extracted from a brown alga has also reported to reduce the *White Spot Syndrome Virus* (WSSV) infection in shrimps [48]. 

Based on previous reports, the present study was conducted to compare and analyze the potential of TWE to improving the prebiotic and immune modulating effects in animals using the model nematode *C. elegans*. In agreement with previous literature, TWE, in the present study, showed greater potential in suppressing secreted virulence factors and toxic metabolites and also reduced the biofilm formation of *P. aeruginosa*, including reduction in its cell density in liquid culture [49,50,51].

It has been reported that *A. nodosum* has many bioactive compounds including polysaccharides, fatty acids, vitamins, antioxidants and peptides [4,8] showing positive effects such as antioxidant, anti-bacterial, cell-mediated immune-modulation and anti-inflammatory activities [11,12]. *A. nodosum* extract have also shown antibacterial properties against many pathogenic microorganisms including *Pseudomonas* [13]. We have recently found that the water extract of Tasco^®^ (TWE) is involved in thermal stress tolerance of *C. elegans* and was also implicated in increasing the life span of the nematode [7]. The present study indicated that the TWE-treatment was able to protect *C. elegans* against *P. aeruginosa* infection by exerting an effect on the innate immunity of the worms as well as through the direct effect on bacterial quorum sensing and virulence factors. Hence, we suggest that the *A. nodosum*-derived extract will have a greater impact in areas such as natural products, nutraceutical, cosmeceutical and perhaps pharmaceutical applications because of the nature of bioactive compounds present in it.

## 4. Experimental Section

### 4.1. Preparation of TWE

Milled *A. nodosum* (Tasco^®^) was a gift from Acadian Seaplants Limited, Dartmouth, Canada. Aqueous extract of Tasco^®^ (TWE) was prepared by extracting 10 g of the product in 40 mL distilled water (DW) at 70 °C for 1 h. The mixture was centrifuged at 10,000 × g for 10 min, at room temperature and the supernatant was transferred into a new glass tube. The pellet was resuspended in 40 mL water and the extraction procedure was repeated. The resulting supernatant was pooled, dried under nitrogen (N_2_) gas and stored at 4 °C until use. A stock solution of 40 mg/mL was made in DW. TWE (300 µg/mL) dissolved in water was used for all the experiments.

### 4.2. Bacterial Strains and Growth Conditions

A clinical pathogenic isolate of *P. aeruginosa* PA14 was a kind gift from Eric Déziel (INRS-Institute Armand-Frappier-Microbiologieet Biotechnologie, Laval, Québec, Canada). Kings B complete medium (with peptone) was used for bacterial culture and maintenance. The following media were used as required in different assays. (i) Modified nematode growth agar medium (0.35% peptone instead of 0.25%) [52]; (ii) LB (tryptone 10 g, yeast extract 5 g, sodium chloride 10 g, final pH 7.0 ± 0.2) [53] broth; (iii) Glycerol alanine minimal medium (10 mL glycerol, 6 g L-alanine, 2 g MgSO_4_, 0.1 g K_2_HPO_4_, 0.018 g FeSO_4_ per liter of medium [54]. All the assays and experiments were repeated with three biological and three technical replicates. For biochemical assays aliquots of bacterial culture was normalized to OD_(620)_ = 1.0 to maintain the equal amount of bacteria in each of the assays.

### 4.3. *C. elegans* Killing Assay

Modified nematode growth medium (0.35% peptone instead of 0.25%) [52] was used in the *C. elegans* slow killing assay. The efficacy of TWE on PA14 induced killing was tested. Treatment plates were prepared by spreading 10 µL of saturated culture of PA14 on the center of the plate. The plate was incubated at 37 °C, for 12 h to establish PA14 lawn. UV killed PA14 and *E. coli* OP 50 plates were used as control. Production of progenies was stopped by treating the worms with 50µM Fluorodeoxyuridine (FuDR) before transferring the worms to assay plates. About 30–40 synchronized L4 worms were transferred to each treatment plate and the number of worms killed was recorded at 4 h intervals by visual observation of plates under microscope. The experiment was carried in three combination of treatments; (i) The synchronized worms maintained on the treatment plates, from their egg stage were exposed to bacterial infection; (ii) The worms, synchronized on plain NGM were infected with a bacterial lawn grown in TWE supplemented culture plates; and (iii) Synchronized worms on their treatment plates were transferred to a bacterial lawn which was grown on TWE supplemented, modified NGM plates. 

### 4.4. Effect of TWE on Immune Response Gene Expression of *C. elegans*

Worms synchronized on TWE supplemented NGM plates were transferred to a pathogen lawn grown on TWE supplemented culture plates; the worms were collected at a young, adult stage, after 18 h of exposure to PA14. Total RNA was extracted from *C. elegans.* The differential expression of immune response genes, as affected by TWE, was studied by quantitative real-time PCR using ABI 7900HT Real-Time PCR system with appropriate primers (Table 1). The following genes: *zk6.7* (*Lypases*), *lys-1* (*Lysozyme*), *spp-1* (*Saponin like protein*), *f28d1.3* (*Thaumatin like protein*), *t20g5.7* (*Matridin SK domain protein*), *abf-1* (*Antibacterial protein*)*and f38a1.5* (*Lectin family protein*) were used in this study [5]. These primers were designed using the online Universal Probe Library Array Design Center. Reverse transcription was performed with 2 μg total RNA for 2 h at 37 °C, using ABI RT system [55]. 

**Table 1:** The immune response primers used in this study.

Sl. No	Primer list	Sequence of oligonucleotides (5′→3′)
1	*ZK6.7-F*	CGAATTCCTCCCAAACAACT
	*ZK6.7-R*	GAATAGGACGTTGTCGCAGA
2	*lys-1-F*	TTCGGATCTTTCAAGAAGGC
	*lys-1-R*	TGGGATTCCAACAACGTAAA
3	*spp-1-F*	TGAACATCGGAACTCTTTGC
	*spp-1-R*	TCAGCTCTTCCTCACACTCG
4	*F28D1.3-F*	AATCTGGATGCCTCGGATAC
	*F28D1.3-R*	CATCTGAGCAGTTGCAGAGC
5	*T20G5.7-F*	ATGTTCTCCCTCAAGACCGT
	*T20G5.7-R*	CGGAAGTGTAAACGACGAAG
6	*abf-1-F*	TGCCTTCTCCTTGTTCTCCT
	*abf-1-R*	ATCCTCTGCATTACCGGAAC
7	*F38A1.5-F*	CTGGGCCGGTATTAATTTGT
	*F38A1.5-R*	GTCTTCTTCGTCACGCACAT
8	*ama-1-F*	CTGACCCAAAGAACACGGTGA
	*ama-1-R*	TCCAATTCGATCCGAAGAAGC

### 4.5. Effect of TWE on *P. aeruginosa* Protease

Protease activity was determined by measuring the ability of culture supernatants to lyse skimmed milk powder. Supernatants from the 18 h culture grown at 37 °C with constant shaking were used for the protease assay. A 100 µL aliquot of *P. aeruginosa* LB culture supernatant, with or without, TWE was added to 900 µL of 0.5% (w/v) skimmed milk in 50 mM Tris HCl (pH 8). The absorbance at OD_(600 nm)_ was measured at 24 and 48 h. The enzyme activity was expressed as OD_(600 nm)_ per µg of protein [56].

### 4.6. Effect of TWE on *P. aeruginosa* Alkaline Protease

Alkaline protease activity of the supernatant from an overnight bacterial culture in LB broth was determined by adding 0.5 mL of supernatant to 1.5 mL of assay buffer (20 mM Tris-HCl, 1 mM CaCl_2_ buffer, pH 8.0) which contained 50 mg of hide remazol blue powder (Sigma). Tubes were incubated at 37 °C for 1 h with constant shaking; the reaction was stopped by placing the tube on ice. The supernatant was removed by centrifuging 5 min at 4000 × g; the absorbance of the supernatant was measured at 590 nm [57].

### 4.7. Effect of TWE on *P. aeruginosa* Elastin

The secreted elastase in the supernatant of PA14 was measured using Congo Red as a substrate [58]. The bacterium was grown in LB broth at 37 °C for 16 h, centrifuged at 15,000 × g at 4 °C for 10 min and 0.5 mL of the supernatant was added to 1 mL of the assay buffer (30 mM Tris buffer, pH 7.2) containing 10 mg of Congo red (Sigma). The mixture was incubated at 37 °C for 6 h with constant shaking, insoluble substrate was removed by centrifugation at 1200 × g for 10 min and absorbance of the supernatant was measured at 495 nm. 

### 4.8. Effect of TWE on *P. aeruginosa* Pyocyanin

Pyocyanin was extracted from a *P. aeruginosa* culture grown in glycerol alanine minimal medium, for 24 h according to the method of Dénervaud *et al.* [59]. The cells were removed by centrifugation and pyocyanin in the supernatant was extracted in chloroform, by mixing 5 mL of supernatant with 3 mL of chloroform. Pyocyanin was then re-extracted into 1 mL of acidiﬁed water (0.2 N HCl) as a pink–red solution. Quantification of the pyocyanin in the solution was obtained from absorbance measured at 520 nm [60]. 

### 4.9. Effect of TWE on the Production of Hydrogen Cyanide in *P. aeruginosa*

Bacterial culture was streaked onto tryptic soya agar medium. Filter paper discs (1.5 cm diameter) were soaked in picric acid solution (2.5 g picric acid, 12.5 g Na_2_CO_3_, and 1 L distilled water) and placed on the upper lids of each Petri dish (after Miller and Higgins, [61]). The dishes were sealed with parafilm and incubated for four days. Hydrogen cyanide (HCN) production was assessed by the presence of a colored zone around the bacterial lawn and the normal yellow of the filter paper turning to a brown to reddish brown color. Reactions were scored as “weak” (*i.e.*, yellow to light brown), “moderate” (*i.e.*, brown) and “strong” (*i.e.*, reddish brown).

### 4.10. Quantification of Hydrogen Cyanide

Bacterial isolates were grown in tryptic soya broth with picric acid solution saturated filter paper strips (10 cm long and 0.5 cm wide) in a hanging position inside the flask at 28 ± 2 °C for 48 h, the sodium picrate in the filter paper was reduced to a reddish compound in proportion to the amount of hydrocyanic acid evolved. The color was eluted by placing the filter paper in a clean test tube containing 10 mL of distilled water and absorbance was measured at 625 nm (after Sadasivam and Manickam, [62]).

### 4.11. Quantification of Siderophore Production

The bacterium was grown in KB broth for 3 days and centrifuged at 2000 rpm for 10 min. The pH of the supernatant was adjusted to 2.0 with HCl and an equal volume of ethyl acetate was added in a separating funnel, mixed well and the ethyl acetate fraction was collected. This process was repeated three times to recover most of the siderophores from the supernatant. The ethyl acetate fractions were pooled, air-dried and dissolved in 5 mL of ethanol (50%). Five mL of ethyl acetate fraction was mixed with 5 mL of Hathway’s reagent (1.0 mL of 0.1 M FeCl_3_ in 0.1 N HCl to 100 mL of distilled water containing 1.0 mL of potassium ferricyanide). The absorbance for dihydroxy phenol was measured at 700 nm [63] using dihydroxy benzoic acid as a standard. The synthesis of siderophores was expressed as µM mL^−1^ of culture filtrate.

### 4.12. RT-PCR Analysis of the Quorum Sensing and Virulence Genes

The relative expressions of quorum sensing genes (*i.e.*, *lasI*, *lasR*, *rhlI* and *rhlR*) and other virulence related genes (*i.e.*, *hcnC*, *aroE*, *rpoN*, *sbe*, *sodB*, *phz*, *pyoS3A*) were analyzed by isolating RNA from the treated and control *P. aeruginosa* (PA14) samples. Primer sequences used are listed in Table 2.

**Table 2:** The PCR primers designed in this study.

Sl. No	Primer list	Sequence of oligonucleotides (5′→3′)
1	*lasI-F*	GCTCCTTGAACACTTGAGCA
	*lasI-R*	GCGCGAAGAGTTCGATAAAA
2	*lasR-F*	CCGCCGAATATTTCCCATA
	*lasR-R*	GATATCGGTTATCTGCAACTGCT
3	*rhlI-F*	GGAGCGCTATTTCGTTCG
	*rhlI-R*	GTCTCGCCCTTGACCTTCT
4	*rhlR-F*	TGCGTTGCATGATCGAGT
	*rhlR-R*	CGGGTTGGACATCAGCAT
5	*hcnC-F*	GCCTGGACAGTTGGTAGGC
	*hcnC-R*	GAACAGAACCTATGACATCGTGA
6	*aroE-F*	TTCTTCGAGCAGGGCAAG
	*aroE-R*	CAATTCGTCCACCAGACGAT
7	*rpoN-F*	ATACCTTCATGCGCAACCA
	*rpoN-R*	GGCTCTGCAGGCTCTTGAT
8	*sbe-F*	CTCGTTGGTCTCCTCGAGTT
	*sbe-R*	CCATCTACCAGCGTGAAGG
9	*sodB-F*	GTTCAAGGAAGAGTTCACCAAGA
	*sodB-R*	GTCGGCCTTCTTCACCAG
10	*16S rRNA-F*	GATTAACGCTTGCACCCTTC
	*16S rRNA-R*	TAAGCACCGGCTAACTTCGT

### 4.13. Bioﬁlm Formation

The bioﬁlm forming ability of the isolates was observed using polystyrene microtitre plates. Brieﬂy, overnight broth cultures in LB broth were diluted to 1:100 into fresh LB broth and then 0.1 mL of the freshly inoculated medium was dispensed into a 96-well polystyrene microtitre plate. The plates were incubated at 37 °C for 8 h without agitation. Bioﬁlm formation was observed by staining the wells with 10 µL of crystal violet [0.1% (w/v) in water]. After the stain was added, the plates were incubated for further 15 min at room temperature and then washed thoroughly with distilled water to remove cells and residual dye. Ethanol (95%) was used to elute any crystal violet from the bioﬁlms and the absorbance of the solubilized dye was measured at 590 nm using a microtitre plate reader (Labsystems Multiskan MS) [64]. 

### 4.14. Testing of Antimicrobial Susceptibility

The disk diffusion method of the Clinical and Laboratory Standards Institute (CLSI), [65] was used to determine the antimicrobial susceptibility of *P. aeruginosa* strains (*n* = 100). 

### 4.15. Statistical Analysis

Significance of the data was analyzed using COSTAT; *p* < 0.05 was considered to be statistically signiﬁcant. Data were analyzed using Fisher’s Least Significant Difference test with *p* ≤ 0.05 using COSTAT statistical software [66].

## 5. Conclusions

Water soluble components of Tasco^®^ reduced *P. aeruginosa* inflicted mortality in the nematode *C. elegans* and at a concentration of 300 µg/mL offered the maximum protection. The improved immune response was associated with induced expression of innate immune response genes. The treatment also altered several virulence components of the *P. aeuroginosa* and lessen the amount of secreted virulence factors. The decline in virulence factors was linked with a significant decline in the expression of regulatory quorum sensing genes involved in quorum sensing. Taken together, our results illustrate that the TWE has the ability to protect the *C. elegans* against *P. aeruginosa* infection by modulating the innate immunity of the worms, besides directly influencing the bacterial quorum sensing and virulence factors.
